# The Mediating Roles of Happiness and Cohesion in the Relationship between Employee Volunteerism and Job Performance

**DOI:** 10.3390/ijerph15122903

**Published:** 2018-12-18

**Authors:** Seunghee Im, Yang Woon Chung, Ji Yeon Yang

**Affiliations:** 1 Department of Business Administration, University of Suwon, 17 Wauan-gil, Bongdam-eup, Hwaseong-si, Gyeonggi-do 445-743, Korea; shim@suwon.ac.kr; 2 Department of Global Business, University of Suwon, 17 Wauan-gil, Bongdam-eup, Hwaseong-si, Gyeonggi-do 445-743, Korea, jiyyang@suwon.ac.kr

**Keywords:** employee volunteerism, happiness, cohesion, in-role behavior, helping behavior

## Abstract

This study investigated the mediating effects of happiness and cohesion in the relationship between employee volunteerism, in-role behavior, and helping behavior. The study surveyed 312 full-time employees in South Korea, and regression analyses and the bootstrapping method were used to test the hypotheses. The study found happiness and cohesion to mediate the relationships between employee volunteerism and in-role and helping behavior. The findings suggest that employee volunteerism can promote a healthy working environment through increased feelings of happiness and cohesion as well as by improving performance behaviors.

## 1. Introduction

Volunteerism is becoming prevalent as it plays an important role in contributing to social welfare [1]. Not only do the beneficiaries of the activity benefit from volunteering, it also helps the volunteer. Literature suggests that volunteering results in better physical and mental health [2,3] and helps to build a healthier society [1]. Since employees spend a significant amount of time and energy at work, the workplace has become an important social context that affects an individual’s well-being. Studies have found that resources devoted to provide a better workplace environment influences an employee’s physical and psychological health [4]. Moreover, studies have found that organizational support and sponsorship of employee participation in corporate volunteering are positively related to the health and well-being of employees [5,6]. 

Corporate volunteering is a method that demonstrates corporate contributions to the social well-being of communities [7]. Accordingly, the importance of employee volunteerism is fast growing in the workplace as it is becoming an essential practice to foster a healthy working environment [8]. Indeed, extant literature has provided empirical support for the importance of employee volunteerism, showing that volunteering is positively associated to various organizational attitudes [9,10] and behaviors [11,12,13,14], as well to the employee’s psychological state [15,16]. 

Despite valuable findings in this field, research has been rather limited as most of the studies have only focused on the direct effects of employee volunteerism on organizational outcomes (e.g., [15]). In order to develop research and comprehensively understand the relationships between volunteerism and organizational outcomes, studies need to further investigate the underlying mechanisms that enable a deeper understanding of the relationships. The conceptual framework of Rodell et al. [17] suggests that several psychological aspects such as identification, morale, and job satisfaction mediate the relationships between volunteerism and workplace behaviors such as task performance and organizational citizenship behavior. In addition, psychological outcomes can link the relationships between employee volunteerism and organizational outcomes [18], as psychological resources accumulated from experiences can have spillover effects from one domain to another domain [19]. 

Therefore, this study examined the mediating effects of employee happiness and cohesion for the relationship between volunteerism and job performance. Job performance has been argued to be multi-dimensional as it includes organizational citizenship behavior, contextual performance, and deviant behavior [20]. Therefore, this study focused on in-role behavior and helping behavior for job performance and contributes to existing literature by empirically testing the relationships between employee volunteerism, happiness, cohesion, in-role behavior, and helping behavior.

## 2. Literature Review and Hypothesis Development

### 2.1. Benefits of Employee Volunteerism

Employee volunteerism can be defined as an employee investing one’s time or skills during a planned activity for a volunteer group under the sponsorship of one’s organization [17,21]. Research in organizational behavior suggests that employee volunteerism has positive effects on workplace attitudes and behaviors [22]. Regarding workplace attitudes, studies have demonstrated that engagement in corporate volunteerism is positively related to morale, organizational pride, trust, identification, commitment, and job satisfaction (e.g., [9,10,23,24,25]). For example, Jones [10] found that employee volunteerism can strengthen and encourage employees to build shared identities with their organization. Similarly, Brockner et al. [9] delineated that corporate volunteerism provides potential for employees to experience self-integrity within their organization, which results in increased levels of organizational commitment. 

Previous research has suggested that participation in corporate volunteerism has positive effects on workplace behaviors. Employee volunteerism has been found to increase task performance and organizational citizenship behavior while decreasing absenteeism and counterproductive behavior (e.g., [11,12,13,14]). When engaging in charitable behaviors, employees are more likely to feel obligated to reciprocate to their organization [26]. In particular, Lavelle [27] argued that volunteering is closely associated to organizational citizenship behavior because they are both considered to be discretionary and deliberate behaviors involving a decision to help others. Gupta and Sharma [28] also suggested that engaged employees in corporate volunteerism exhibited citizenship behaviors.

Furthermore, volunteering leads to positive psychological states. Research has showed that volunteerism is related to enhanced self-esteem, excitement, enthusiasm, happiness, and positive emotions [3,15,16,29]. The psychological needs perspective provides justification for the positive psychological effects of volunteerism. According to this perspective, volunteerism meets an individual’s needs for self-expression and approval from others, and fulfills personal obligations. By fulfilling these needs, self-esteem increases which further results in other positive psychological consequences [30]. In addition, Kahn [5] suggested that activities outside of work such as volunteering can charge employees and provide them with more psychological resources. Similarly, Pajo and Lee [7] argued that employees can experience positive psychological reactions through volunteering and generate enthusiasm.

### 2.2. Hypotheses Development

Volunteering has been argued to be an important determinant of happiness [15]. Prior studies have found volunteering to be beneficial to well-being as it contributes to decreased psychological distress and depression while increasing self-esteem, life satisfaction, and physical health (e.g., [3,31,32]). From a psychological point of view, volunteering enhances self-esteem and confidence, and gives meaning to life, which in turn fosters happiness [33]. Subsequently, research has found volunteering to enhance happiness. Borgonovi [2] indicated that people who volunteer report greater happiness than people who do not volunteer. Pajo and Lee [7] reported that volunteers experience considerable enjoyment and satisfaction when working with others in volunteer activities. In addition, Nadeem [34] argued that happy individuals are more likely to help others and that happiness is related to volunteering, thus suggesting a cyclical relationship between volunteering and happiness.

Studies have found volunteerism to have positive effects on task performance and organizational citizenship behavior (e.g., [10,27]). Employees who value volunteering will be more likely to positively perceive their organization which in turn promotes positive workplace behaviors. In addition, Gilder et al. [22] argued that volunteering increases positive attitudes about the volunteer work and other volunteers, thus increasing organizational citizenship behavior and other work-related behaviors. In this regard, volunteering should be related to in-role behavior and helping behavior. In-role behavior refers to expected behaviors specified by one’s role and is the basis of regular and ongoing job performance [35,36]. Helping behavior refers to prosocial behaviors that reflect authentic concern and courtesy toward coworkers [35,37].

Recent research shows that happiness is important not only for an employee’s own betterment but also for one’s organization [38]. People are more likely to be more active in helping others when they experience positive emotions [39]. Positive moods help promote the value of a target object which allows an individual to recall positive information and experiences which can then result in prosocial behavior [40]. In contrast, individuals that perceived negative moods such as feelings of alienation and psychological suffering were more likely to be dissatisfied and angered, and reciprocated by engaging in negative behaviors such as self-abasement and intentionally decreasing efforts [41]. 

Although different in social context, the relationships between volunteering, happiness, in-role behavior, and helping behavior can be supported by affective events theory. Affective events theory explains how work events affect an employee’s moods and emotions which then influences work attitudes and behaviors [42]. Although corporate volunteering is not within the organizational context but can be considered to be a work-related event, volunteering can trigger emotions such as happiness and have spillover effects on work behaviors such as in-role behavior and helping behavior. Based on understanding the relationships between employee volunteerism, happiness, and workplace behaviors, we can predict happiness to mediate the relationships between employee volunteerism with in-role behavior and helping behavior. Thus, we hypothesize the following:

**Hypothesis** **1** **(H1).** *Happiness will mediate the relationships between employee volunteerism with in-role behavior and helping behavior.*

Volunteerism can affect perceptions on interpersonal relationships [43]. When individuals work interdependently across personal differences, they appreciate other’s knowledge, experiences and efforts, which then facilitate individual bonding [44]. Humphrey et al. [45] claimed that volunteering provides opportunities to interact and collaborate with colleagues in a team-based environment which allows for favorable interpersonal relationships. In turn, these opportunities build up camaraderie, which affects attitudes towards the team. Therefore, employee volunteerism allows for congenial interactions with other organizational members and increases the chance to build relationships and reinforce companionship ties within the organization. As a result, employee volunteerism generates strong connections that create trust within the group, thereby fostering cohesion among employees [45,46].

According to social identity theory, individuals interpret their identities through interactions with others in various social contexts [47]. The social identity perspective suggests how an individual identifies himself or herself with others which then affect outcomes such as group cohesion and cooperation [48,49]. When an individual identifies with a group, the values and practices of the group become more salient, thereby emphasizing conformity to group norms and similarity in attitudes and behavior [50]. Furthermore, corporate social responsibility (CSR) literature suggests that corporate volunteerism can enhance an employee’s pride in belonging to a socially desirable organization and boost an employee’s desire to identify with the organization according to the social identity perspective [10]. Therefore, employees who identify with their organization are likely to exhibit enhanced work performance due to a strong sense of belongingness to their organization and having a tendency to be motivated to achieve organizational goals and exert a significant amount of effort [51]. 

A central aspect of cohesion is the feeling of being accepted and all forces acting on team members belonging to the team [52]. Cohesion is fundamental for quality related teamwork outcomes such as work performance and helping behavior (e.g., [53,54,55]). In particular, researchers have focused on the role that positive affect influences the motivational processes of individual and team outcomes [56]. For example, Beal et al. [57] meta-analysis has found evidence of a positive link between cohesion, work performance, and helping behavior.

Helping behaviors are more likely to occur when individuals have strong cohesive feelings [57], especially because cohesive members are more committed to each other. Cohesion further promotes one’s responsiveness to team members and the team [58,59]. In addition, cohesiveness increases the reciprocity between colleagues as they respect each member’s contribution of ideas, values, and assistance to other members. Therefore, this suggests that cohesion is positively related to helping behavior and considering the relationship between employee volunteerism, cohesion, in-role behavior, and helping behavior; it is natural to posit that cohesion will mediate the relationships between employee volunteerism with in-role behavior and helping behavior. Hence, we propose following:

**Hypothesis** **2** **(H2).** *Cohesion will mediate the relationships between employee volunteerism with in-role behavior and helping behavior.*

## 3. Methodology

### 3.1. Data Collection 

Full-time employees in South Korea were surveyed using a self-reported questionnaire. We selected companies that engaged in numerous employee volunteering programs that encouraged employees to voluntarily participate. Companies in a wide range of industries were included in the study in order to ensure heterogeneity of the respondents. The questionnaires were mailed to volunteering program managers for each company. The questionnaire was given to each respondent from the manager and the questionnaire included the instructions of the study and measurement items. We stressed anonymity and confidentiality of the survey to reduce social desirability bias by placing each survey in individual envelopes.

In total, 312 completed questionnaires were collected. The average age of the respondents was 34.048 years (S.D. = 8.146) and average tenure was 8.961 years (S.D. = 7.434). In total, 53.205% were male and the majority of the respondents (84.47%) had at least a college degree. Organizational position covered entry level (47.76%) and managers (52.24%). In terms of industry, respondents were from financial services (40.75%), manufacturing (32.29%), retail (16.93%), hospitality (7.52%), and other industries (2.51%).

### 3.2. Measurement

As the measures were originally developed in English, translation and back-translation procedures recommended by Brislin [60] were used to validate the measures. All items were measured with a seven-point Likert scale ranging from ‘strongly disagree’ to ‘strongly agree’. 

Employee volunteering was measured with three items [11]. Sample items included: ‘I give my time to help a volunteer group’ and ‘I engage in activities to support a volunteer group.’ The reliability of this sale was 0.934.

Happiness was measured with three items based on Lyubomirsky and Lepper’s [61] scale. Sample items included, ‘In general, I consider myself happy’ and ‘Compared to most of my peers, I consider myself happy.’ The reliability of this sale was 0.940.

Cohesion was measured with five items adapted from Wech et al. [62]. Sample items included: ‘There is a high sprit of teamwork among my coworkers’ and ‘The people I work with make my job easier by sharing their ideas and opinions with me.’ The reliability of this sale was 0.930.

In-role behavior was measured with five items adapted from Williams and Anderson’s [63] scale. Sample items included: ‘I adequately complete assigned my duties’ and ‘I perform tasks that are expected.’ The reliability of this sale was 0.960.

Helping behavior was measured with seven items [58]. Sample items included: ‘Help others who have been absent,’ and ‘Takes a personal interest in other employees.’ The reliability of this sale was 0.922.

We also measured gender, level of education, age, organizational tenure, and positive affect as control variables. Positive affect was measured with four items based on the scale of Watson et al. [64]. Sample items include: ‘enthusiastic,’ and ‘excited.’ These variables were controlled for all of the analyses. 

## 4. Results

Table 1 presents the means, standard deviations, and zero-order correlations for the study. 

Confirmatory factor analysis (CFA) was performed to ensure the validity of the measurements. The measurement model comprising of all indicators was examined to validate discriminant validity [65]. As shown in Table 2, the hypothesized five-factor model (χ^2^ (171) = 343.276, *p* < 0.01; normal fit index (NFI) = 0.952, Tucker-Lewis Index (TLI) = 0.970, comparative fit index (CFI) = 0.975, root mean square error of approximation (RMSEA) = 0.057) yielded a good fit to the data compared to the other models. Across the measurement models in the study, all standardized factor loadings were significant (*p* < 0.01) with the lowest standardized loading equal to 0.805. 

To assess the convergent validity, composite reliability (CR) and the average variance extracted (AVE) were computed for each variable. The composite reliability coefficients exceeded the recommend value of 0.70 for all constructs ranging from 0.832 to 0.969 and the average variance extracted values for the constructs were all greater than the recommended value of 0.50 ranging from 0.693 to 0.855. The values of AVE were also compared with the squared variable correlations and the results show that all AVEs were higher than any squared correlation values, further supporting discriminant validity [66]. Thus, the results indicated that the constructs for the model had sufficient reliability and validity.

To test the hypothesized model, the conditions of mediation were examined with structural equation modeling (SEM). To assess mediation, the fit of several mediation models was compared to the hypothesized model. In addition, the Chi-squared difference test was conducted to determine if significant differences existed in the overall fit among the models. As depicted in Table 3, the results indicated that the hypothesized full mediated model (Model 1 (χ^2^ (171) = 343.276, *p* < 0.01; NFI = 0.952, TLI = 0.970, CFI = 0.975, RMSEA = 0.057)) significantly fit the data better than Model 2 (△χ^2^ = 60.892, *p* < 0.001), which was a partial mediation model. Also, Model 1 resulted in a better fit compared to Model 3 with a direct path from volunteering to in-role behavior (△χ^2^ = 71.471, *p* < 0.001). Further, Model 1 fit the data significantly better than the Model 4, where there was a direct path from volunteering to helping behavior (△χ^2^ = 61.498, *p* < 0.001). Thus, the results indicated that Model 1 is significantly better than the other models. 

**Figure 1:**
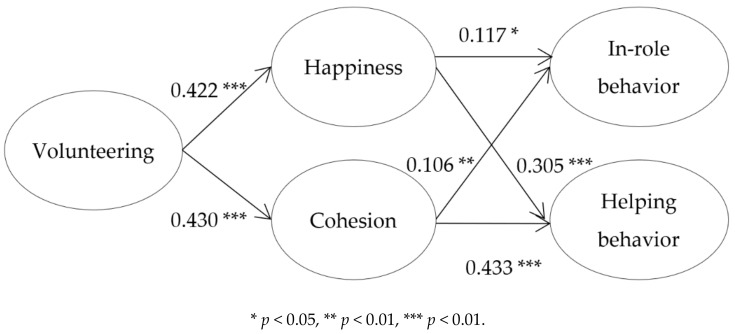
Estimates of the structural mediation model.

The structural coefficients for the hypothesized mediation model are displayed in Figure 1. The results show that volunteering was positively related to happiness (*β* = 0.422, *p* < 0.001) and cohesion (*β* = 0.430, *p* < 0.001). In addition, happiness was positively associated with in-role behavior (*β* = 0.117, *p* < 0.05) and helping behavior (*β* = 0.305, *p* < 0.001). Further, cohesion was positively related to in-role behavior (*β* = 0.106, *p* < 0.01) and helping behavior (*β* = 0.433, *p* < 0.001).

We further conducted bootstrapping tests to confirm multiple mediation [67]. This approach makes it possible to avoid power problems introduced by asymmetric and other non-normal samplings of an indirect effect through the application of bootstrapped confidence intervals [68]. The bootstrapping technique was performed with 5000 samples at 95% confidence intervals to assess the multiple mediation mechanisms. Hypothesis 1 predicted that happiness will mediate the relationships between volunteering with in-role and helping behavior. As presented in Table 4, volunteering was positively related to happiness (*β* = 0.156, *p* < 0.01). In addition, happiness was found to be positively related to in-role behavior (*β* = 0.121, *p* < 0.05) and helping behavior (*β* = 0.138, *p* < 0.01). Furthermore, Table 5 and Table 6 show that volunteering has an indirect effect on in-role behavior and helping behavior through happiness. The bootstrap results with a bootstrapped 95% CI around the indirect effects did not contain zero for in-role behavior (0.019, 0.113) and helping behavior (0.002, 0.049). In sum, happiness was found to fully mediate the relationship between volunteering and in-role behavior while happiness partially mediated the relationship between volunteerism and helping behavior as there was a significant direct effect to helping behavior; thus Hypothesis 1 was supported. 

Hypothesis 2 posited that cohesion will mediate the relationship between volunteering and in-role behavior and helping behavior. As shown in Table 4, volunteering was found to be positively related to cohesion (*β* = 0.149, *p* < 0.01). Further, cohesion was found to be positively related to in-role behavior (*β* = 0.289, *p* < 0.001) and helping behavior (*β* = 0.444, *p* < 0.001). Table 5 and Table 6 indicate that cohesion had a positive impact on in-role behavior (*β* = 0.289, *p* < 0.001) and the bootstrap results with a bootstrapped 95% CI around the indirect effects did not contain zero for in-role behavior (0.010, 0.086). Further, cohesion was found to mediate the relationship between volunteering and helping behavior as the bootstrapped 95% CI around the indirect effect did not contain zero (0.015, 0.124). Taken together, cohesion was found to fully mediate the relationship between volunteering and in-role behavior. For helping behavior, cohesion partially mediated the relationship as there was a significant direct effect to helping behavior, therefore Hypothesis 2 was supported. 

## 5. Discussion

The purpose of the study was to identify the relationships between employee volunteerism, happiness, cohesion, and job performance. In particular, the study aimed to investigate the mediating effects of happiness and cohesion on the relationships between employee volunteerism and in-role behavior and helping behavior. Consistent with prior research that has found that volunteerism to affect an individual’s psychological state (e.g., [15]) and workplace behaviors (e.g., [10]), the study findings confirm that employee volunteerism is positively associated with happiness, cohesion, in-role behavior, and helping behavior. Furthermore, based on affective events theory and social identity theory, the results also showed that both happiness and cohesion mediated the relationships between employee volunteerism with in-role behavior and helping behavior to further explain the process by which volunteerism influences psychological states and workplace behaviors. 

Our theoretical contribution lies in extending extant knowledge on employee volunteerism and workplace behavior by demonstrating multiple mediation. Prior studies primarily focused on the effects of volunteerism in either psychological (e.g., [16]) or organizational behavior aspects (e.g., [11]) and examined the direct effect of volunteerism (e.g., [13]). By presenting an integrative framework to explain the mediating effects of happiness and cohesion for the relationships between volunteerism, and in-role behavior, and helping behavior, our study contributes to a better understanding of how employee volunteerism influences workplace behaviors through psychological states. Moreover, if volunteerism is related to employee cohesion and happiness, other organizational outcomes associated to these variables may also be significantly related to employee volunteerism. Happiness literature reveals that happiness precedes numerous positive outcomes as well as behaviors that parallel success [69]. Likewise, cohesion is an important construct that induces various attitudinal and behavioral outcomes such as morale, job satisfaction, problem solving ability, and quality of life (e.g., [70]). Thus, the study findings provide a theoretical basis for research moving forward by suggesting that employee volunteerism may have indirect effects on other workplace attitudes and behaviors beyond in-role behavior and helping behavior. Moreover, as there are limited studies on volunteering and job performance [17], thus this study further confirms the relationship.

From a management perspective, our findings offer managerial insights involved in designing and implementing employee volunteering programs. This research suggests that the strategic value of corporate volunteerism highlights the important role of employee volunteerism on influencing positive organizational behaviors through positive psychological states. As mentioned above, happiness and cohesion may have a positive impact on a variety of organizational outcomes. Consequently, employee volunteerism can be used as a useful strategic tool to create favorable organizational outcomes by positively affecting the psychological states of organizational members. Therefore, the findings of the study provide a basis for motivating managers to invest in volunteering programs for both employees and the organization. 

The study has some limitations to be mentioned as with any study. First, according to the findings of the structural equation model, happiness seems to strongly relate to in-role behavior while cohesion seems to strongly relate to helping behavior. Happiness is a subjective perception of one’s inner state and it is fundamentally related to personal outcomes [71]. In particular, happiness enhances identification with an employee’s roles in the workplace [38]; therefore, happiness may have a stronger impact on in-role behavior than on helping behavior. In contrast, a key facet of cohesion is the feeling in relationships with others [52] as it is conceptually related to interpersonal outcomes. In the work context, cohesion fosters the interactions between co-workers [58]. Thus, it is natural that cohesion has a stronger effect on helping behavior rather than on in-role behavior. However, there is a need for caution in these interpretations, as the study did not permit empirical comparisons of happiness and cohesion. Therefore, future research should provide more theoretical frameworks and empirical multiple mediation comparisons which allow for a better understanding of the mediation processes. 

Second, a potential limitation of the study is the generalizability of the findings. The results may not be generalized because this study was conducted in South Korea. In terms of corporate social responsibility, recent research has identified cross-cultural differences between countries or cultures regarding CSR perceptions and organizational responses. For example, Scott et al. [72] suggested that perceptions of corporate volunteerism are influenced by national culture. Accordingly, culture should be considered as a pertinent factor when implementing employee volunteerism [73]. Cultural differences are also important considerations when understanding the relationships between constructs in happiness (e.g., [74]) and cohesion literature (e.g., [75]). Therefore, research that replicates our findings in other cultural contexts would be meaningful in order to further validate our results. In addition, future research should examine how national and cultural factors are related to the impact of employee volunteerism on organizational outcomes from the perspective of cross-cultural studies. 

Lastly, although the study predicted causality based on theories, it is possible to generate alternative explanations to explain the relationship between employee volunteerism, happiness, cohesion and job performance due to the cross-sectional nature of the study. For example, Wang and Graddy [76] suggested that happiness leads to volunteering. Hence, further research using time-lagged longitudinal designs would be constructive in providing a more robust evidence within causality. 

## 6. Conclusions

Employee volunteerism is a rapidly growing topic in both practical and academic domains. However, in spite of valuable research efforts showing positive effects of employee volunteerism and organizational outcomes, previous studies have not comprehensively explained the underlying mechanisms between volunteerism and workplace behaviors. Therefore, this study identified the mediating effects of happiness and cohesion on the relationships between employee volunteerism and in-role behavior and helping behavior. Findings of the study suggested that employee volunteerism induces happiness and cohesion, which in turn promotes individuals to engage in-role behavior and helping behavior, thereby supporting the mediating roles of happiness and cohesion. This research also contributes theoretically in that it expands extant literature on volunteerism by demonstrating the relationships between employee volunteerism, happiness, cohesion, in-role behavior, and helping behavior. Further, findings of the study can be applied to corporate management in creating a healthy working environment through the strategic use of employee volunteerism programs. 

## Figures and Tables

**Table 1:** Descriptive statistics and zero-order correlations.

Variable	Mean	SD	1	2	3	4	5	6	7	8	9	10
1. Gender	1.468	0.500										
2. Education	2.625	0.880	−0.483 **									
3. Age	34.048	8.146	−0.432 **	0.270 **								
4. Position	1.933	1.134	−0.381 **	0.291 **	0.644 **							
5. Tenure	8.961	7.434	−0.097	−0.076	0.818 **	0.517 **						
6. PA	5.096	1.033	−0.129 *	0.122 *	0.088	0.125 *	0.055					
7. EV	5.141	1.139	−0.005	0.012	0.068	0.022	0.064	0.426 **				
8. Happiness	5.385	1.065	−0.210 **	0.083	0.200 **	0.181 **	0.149 **	0.606 **	0.398 **			
9. Cohesion	5.267	1.049	−0.228 **	0.097	0.157 **	0.207 **	0.133 *	0.595 **	0.369 **	0.654 **		
10. IRB	5.444	0.900	−0.193 **	0.158 **	0.246 **	0.199 **	0.182 **	0.524 **	0.302 **	0.521 **	0.580 **	
11. HB	5.395	0.928	−0.169 **	0.069	0.172 **	0.194 **	0.127 *	0.605 **	0.429 **	0.639 **	0.752 **	0.616 **

PA: positive affect; EV: employee volunteerism; IRB: in-role behavior; HB: helping behavior; * *p* <0.05, ** *p* <0.01.

**Table 2:** Fit statistics for measurement models.

Models	χ^2^	df	NFI	TLI	CFI	RMSEA
One-factor model	2660.363	181	0.628	0.586	0.643	0.210
Two-factor model ^a^	1962.522	180	0.726	0.701	0.744	0.178
Three-factor model ^b^	1396.984	178	0.805	0.793	0.825	0.148
Four-factor model ^c^	855.115	175	0.881	0.883	0.902	0.112
Five-factor model ^d^	343.276	171	0.952	0.970	0.975	0.057

**Notes**: ^a^ = all constructs combined into one factor; ^b^ = In-role behavior, helping behavior, happiness; and cohesion combined into one factor; ^c^ = In-role behavior and helping behavior combined into one factor, happiness and cohesion combined into one factor; ^d^ = In-role behavior and helping behavior combined into one factor. df = degree of freedom; NFI = normal fit index; TLI = Tucker-Lewis Index; CFI = comparative fit index; RMSEA = root mean square error of approximation.

**Table 3:** Results of model comparisons.

Models	χ^2^	df	NFI	TLI	CFI	RMSEA	∆χ^2^
Model 1	343.276	171	0.952	0.970	0.975	0.057	-
Model 2	404.168	176	0.944	0.961	0.967	0.065	60.892
Model 3	414.747	177	0.942	0.959	0.966	0.066	71.471
Model 4	404.774	177	0.943	0.961	0.967	0.064	61.498

**Notes**: Hypothesized model 1(hypothesized full mediation model); Alternative model 2 (partial mediation model); Alternative model 3 (volunteering → in-role behavior included); Alternative model 4 (volunteering → helping behavior included). The Chi-squared differences from Model 1. df = degree of freedom; NFI = normal fit index; TLI = Tucker-Lewis Index; CFI = comparative fit index; RMSEA = root mean square error of approximation.

**Table 4:** Multiple mediators regression analysis.

Variables	Happiness		Cohesion	
	Coefficient	SE	Coefficient	SE
Volunteering	0.156 **	0.047	0.149 **	0.047
	In-role behavior		Helping behavior	
	Coefficient	SE	Coefficient	SE
Volunteering	0.021	0.040	0.094 **	0.033
Happiness	0.121 *	0.054	0.138 **	0.044
Cohesion	0.289 ***	0.054	0.444 ***	0.044
	R^2^ = 0.433 F = 28. 502 ***		R^2^ = 0.633 F = 64.317 ***	

* *p* < 0.05, ** *p* < 0.01, *** *p* < 0.001. SE = standard error.

**Table 5:** Indirect effects of volunteering on in-role behavior.

Indirect Effects of Volunteering on In-Role Behavior	Bootstrapping Percentile 95 percent CI			
	Point Estimate	SE	Lower	Upper
*Indirect effects*				
Happiness	0.019	0.024	0.019	0.113
Cohesion	0.043	0.019	0.010	0.086
Total	0.062	0.024	0.019	0.113

Notes: CI = confidence interval; Bias-corrected bootstrapping results: 5000 bootstrap samples. SE = standard error.

**Table 6:** Indirect effects of volunteering on helping behavior.

Indirect Effects of Volunteering on Helping Behavior	Bootstrapping Percentile 95 percent CI			
	Point Estimate	SE	Lower	Upper
*Indirect effects*				
Happiness	0.021	0.012	0.002	0.049
Cohesion	0.066	0.028	0.015	0.124
Total	0.088	0.033	0.024	0.156

Notes: CI = confidence interval; Bias-corrected bootstrapping results: 5000 bootstrap samples. SE = standard error.
